# Serum cortisol and testosterone levels in idiopathic central serous chorioretinopathy

**DOI:** 10.4103/0301-4738.57143

**Published:** 2009

**Authors:** Shaik M Zakir, M Shukla, Zaka-ur-rab Simi, J Ahmad, Mahmood Sajid

**Affiliations:** Retina Service, Institute of Ophthalmology, Aligarh Muslim University, Aligarh, India.; 1 Division of Endocrinology, Department of Medicine, Jawaharlal Nehru Medical College, Aligarh Muslim University, Aligarh, India.

**Keywords:** Cortisol, idiopathic central serous chorioretinopathy, testosterone

## Abstract

**Context:**

The preferential occurrence of idiopathic central serous chorioretinopathy (ICSC) in males with a typical Type A personality and behavior and a relative absence in females is a possible indicator towards the role of serum cortisol and /or the male sex hormone testosterone.

**Aims:**

To study levels of cortisol and testosterone in ICSC.

**Settings and Design:**

Case-control study in a tertiary care teaching hospital.

**Materials and Methods:**

The study was conducted on 23 cases of ICSC. Twelve patients with unilateral sudden painless loss of vision of less than one month duration served as controls. Serum cortisol and testosterone levels at 8.00 a.m. were estimated by radioimmunoassay in both groups.

**Statistical analysis used:**

Statistical analysis was done using SPSS 13.0 software. Independent Sample t-test was applied to analyze statistical significance between the two groups.

**Results:**

Mean age of patients with ICSC was 37.1 ± 9.7 years and 96% of the patients were males. Mean serum cortisol levels were significantly higher (*P*=0.002) in patients with ICSC i.e., 495.02 ± 169.47 nano moles/liter (nmol/L) as compared to controls i.e., 362.25 ± 51.54 nmol/L. Mean serum testosterone levels were 3.85 ± 1.81 nano grams/ml (ngm/ml) and 4.23 ± 1.89 ngm/ml in cases and controls respectively and the difference was not statistically significant (*P*=0.58).

**Conclusions:**

ICSC is associated with elevated 8.00 a.m. serum cortisol levels. However, mean serum testosterone levels in both patients of ICSC and controls were within normal range.

Von Graefe[1] in 1866 first described recurrent serous detachment of macula and named it ‘recurrent central retinitis’ and subsequently various other terms like idiopathic flat detachment of macula, and central serous retinopathy were used.[2] Maumenee[3] on fundus fluorescein angiography (FFA) noted that the macular detachment resulted from a leak in the retinal pigment epithelium (RPE). Gass[4] classically described the condition and named it idiopathic central serous chorioretinopathy (ICSC). It is characterized by accumulation of transparent fluid below the neurosensory retina causing a circumscribed macular detachment. In 94% of cases, the fluid accumulates under the neurosensory retina (Type I), in 3%, the RPE alone is detached (Type II) and in the rest both neurosensory retina and RPE are elevated (intermediate type).[5]

The etiology of ICSC still remains unclear and numerous hypotheses have been put forward. The choriocapillaries and RPE are basically involved and various anatomical and functional changes affecting these important structures have been demonstrated. ICSC has been associated with Type A personality,[2] elevated endogenous cortisol[6] and corticosteroid therapy in various forms.[7] This condition, predominantly, affects young males and its incidence declines with advancing age. As plasma testosterone levels also decline with age,[8] it is possible that testosterone may have an important role in predisposing males to ICSC. The present work was, therefore, undertaken to study the levels of cortisol and testosterone in ICSC.

## Materials and Methods

The present study was conducted in a tertiary care teaching hospital between January 2006 and September 2007. The study was approved by the local ethical committee of the institution and all efforts were made to remain true to the guidelines of the Declaration of Helsinki. The study group comprised 23 patients with recent onset of painless diminution of vision due to ICSC. Twelve age and sex-matched patients with acute unilateral, sudden, painless loss of vision of recent onset, due to various causes were included in the control group. Since sudden loss of vision is a stressful event, its occurrence could result in elevated cortisol levels in any subject. The inclusion of patients with an acute unilateral, sudden, painless loss of vision of recent onset as controls obviated the possibility of bias on this account. Informed consent was taken from all patients.

A detailed history of the visual disturbance, its onset, duration and progression, similar complaints in the past, drug intake, (specially use of corticosteroids/ sex steroid hormones in any form) was elicited. Patients who had taken corticosteroids/sex steroids in any form (topical eye drops, skin creams, intranasal/ inhalational sprays; systemic steroids like oral, intravenous/ intramuscular) in the last one month were excluded from the study. Patients with any other ocular or systemic disease, any surgery or trauma within one month of presentation, alcohol abuse or dependence, and major depression were excluded from our study, since all these conditions can independently alter the endogenous cortisol levels.

Examination of visual acuity, refractive status, and slit-lamp biomicroscopy (including +90 D) was done. FFA was done to confirm the diagnosis and localize the site of leakage. Estimation of 8.00 a.m. serum cortisol was done in all patients, and testosterone in male patients only by radioimmunoassay (RIA) using the kit supplied by DiaSorin Inc. 1951 Northwestern Avenue P.O. Box 285 Stillwater, MN 55082-0285 U.S.A. following the technique of Collins *et al*.[9]

## Results

The study group comprised 23 cases with ICSC, of whom 22 (96%) were males. The control group had 11 males and one female. Of the patients in the control group, 10 had retinal detachment (cases), and one each had central retinal artery occlusion and vitreous hemorrhage. The mean age was 37.1±9.7 years in patients with ICSC and 35.7±9.2 years in controls. Mean weight of patients in the study group was 53.80±5.44 Kg and in the control group was 51.87±6.95 Kg. The mean duration of visual disturbance at presentation was 3.59±1.73 weeks, and 74% presented within two to four weeks. FFA showed leakage of the dye in the following patterns: single leak in 17 (74%), and multiple leaks in six (26%) eyes.

**Graph 1:**
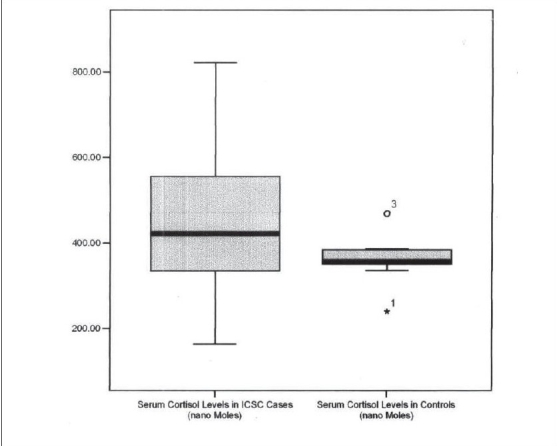
Box chart showing serum cortisol levels in idiopathic central serous chorioretinopathy (ICSC) cases and controls

The mean 8.00 a.m. serum cortisol was 495.02±169.47 nmol/L in cases and 362.25±51.54 nmol/L in controls and this difference between cases and controls was statistically significant (*P*=0.002) [Graph 1]. Table 1 shows the serum cortisol levels in ICSC cases and controls. Two out of 23 cases (9%) had high cortisol values while nine (39%) cases had high borderline levels (Normal range 193 - 690 nmol/L). There was no correlation between cortisol levels and duration of symptoms (r = −0.032, *P* = 0.137).

**Table 1:** Serum cortisol levels in idiopathic central serous chorioretinopathy (ICSC) cases and controls

Serum cortisol levels (nmol/L)	Cases No. (%)	Controls No. (%)
00 - 99	0 (0)	0 (0)
100 - 199	1 (4.35)	0 (0)
200 - 299	2 (8.70)	1 (8.33)
300 -399	1 (4.35)	10 (83.33)
400 - 499	7 (30.43)	1 (8.33)
500 - 599	6 (26.08)	0 (0)
600 - 699	4 (17.39)	0 (0)
700 - 799	0 (0)	0 (0)
800 - 899	2 (8.70)	0 (0)
Total	23 (100)	12 (100)

Of the 11 cases having cortisol levels higher than mean value of 495.02 nmol/L, seven had a single leak and four had multiple leaks. Seven of the 17 cases with a single leak had higher and 10 cases had lower than mean cortisol levels.

**Graph 2:**
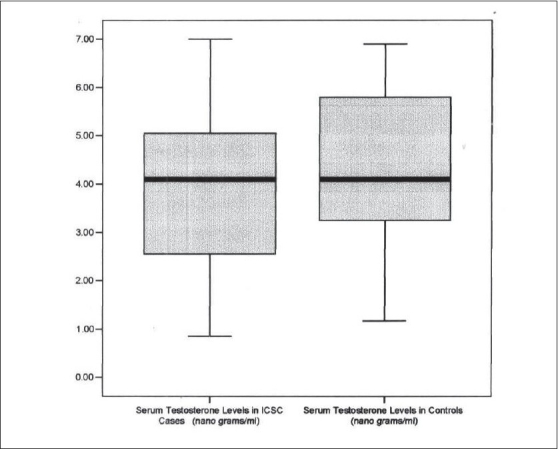
Box chart showing serum testosterone levels in idiopathic central serous chorioretinopathy (ICSC) cases and controls

The mean serum testosterone level in cases was 3.85±1.81 ngm/ml which was less than the mean serum testosterone in controls 4.23±1.89 ngm/ml but this difference was not statistically significant (*P*=0.58) [Graph 2]. Seven of 22 (32%) cases (one female excluded) and only two out of 11 controls had testosterone levels below normal range (Normal range 2.8 - 9.0 ngm/ml). Among the ICSC cases no patient had high testosterone levels [Table 2]. Of the 12 cases with higher than mean testosterone levels, eight had single and four had multiple leakage pattern, whereas all three cases of diffuse leakage had testosterone above mean levels. Out of 17 cases with a single leak, testosterone levels were higher than mean in eight cases and lower in nine cases. There was no correlation between testosterone levels and number or pattern of leakage.

**Table 2:** Serum Testosterone Levels in idiopathic central serous chorioretinopathy (ICSC) Cases and Controls

Serum Testosterone Levels (nmol/L)	Cases No. (%)	Controls No. (%)
0 - 0.99	1 (4.55)	0 (0)
1 - 1.99	2 (9.09)	2 (18.18)
2 - 2.99	4 (18.18)	0 (0)
3 - 3.99	4 (18.18)	3 (27.28)
4 - 4.99	6 (27.27)	2 (18.18)
5 - 5.99	1 (4.55)	2 (18.18)
6 - 6.99	2 (9.09)	2 (18.18)
7 - 7.99	2 (9.09)	0 (0)
Total	22 (100)	11 (100)

## Discussion

Maculopathy resembling ICSC has been reported in a patient receiving glucocorticoids for reactive arthritis.[10] There are reports of initiation and worsening of ICSC in patients under systemic steroid treatment for other reasons.[11,12] In many cases there was a close temporal relationship between use of corticosteroids and development of ICSC; exacerbations or remissions of the symptomatology correlated with the glucocorticoid levels as well.[13]

Glucocorticoids promote blood coagulation causing choroidal hypoperfusion, affect the production of prostaglandins and, therefore, may affect the regulation of choroidal blood flow. Glucocorticoids inhibit collagen formation (a main component of Bruch's membrane), alter ion and water transport of epithelia primarily through the mineralocorticoid receptors. Cortisol may also directly damage the RPE cells or their tight junctions and may delay any reparative process in damaged RPE cells by suppressing the synthesis of extracellular matrix components and inhibiting fibroblastic activity.

ICSC frequently occurs in highly ambitious people who are stressed and overworked. Yannuzzi[2] has given a detailed description of the association of ICSC with male sex, middle age, ‘stress’ and Type A personality, the features of which are competitive drive, a sense of urgency, an aggressive nature, a hostile temperament, intense and sustained drive to achieve, state of restlessness, perception of all responsibilities with an element of challenge, extraordinary mental and physical alertness, desire for recognition and advancement, frequent involvement with deadlines and multiple simultaneous tasks. Physiological changes associated with Type A personality are increased pupillary size,[14] increase in skin temperature[15] and pulse rate,[15] blood pressure,[16] platelet aggregation,[17] increased plasma cortisol,[6,18] plasma epinephrine[19] and norepinephrine,[20] increased urinary excretion of cortisol[19] and epinephrine,[19] increased urinary excretion of norepinephrine[21] and testosterone.[8]

Various studies show a higher prevalence in men ranging from 72%[22] to 88%[23] with a peak around 45 years in men and higher in women though no age is immune. In our study, the age of patients ranged from 20 to 49 (mean 33.7±9.2) years with 96% patients being males.

There are isolated reports of association of ICSC with raised serum cortisol levels.[6,24] These observations assume significance and point towards a relationship between endogenous levels of cortisol and development of ICSC. In the present study statistically significant higher mean serum cortisol levels were seen in ICSC cases than controls. This throws some light on the possible effects of cortisol (on Bruch's membrane and choriocapillaries) and development of ICSC in young men. This is an interesting observation and we thought that there might be some correlation between duration of ICSC and levels of serum cortisol. However, no significant correlation between the two was observed as the cortisol levels were not found to be significantly higher in those with longer duration of ICSC. We also thought that higher levels of cortisol could possibly cause more damage to choriocapillaris, Bruch's membrane and RPE complex, thereby presenting with multiple/ smokestack leaks. Out of the 11 cases where serum cortisol was towards the higher limit of normal, only a single leak was observed in seven cases and multiple leaks in four cases.

Androgens or testosterone have never been directly implicated as a factor in the pathogenesis of ICSC. The higher incidence of ICSC in young males and a gradual decline as the age advances correlates with the levels of plasma testosterone which also decline with age in older men.[25] The preferential occurrence of ICSC in males with a typical Type A personality and behavior and a relative absence in females is a possible indicator towards the role of the male sex hormone testosterone. The female sex hormones estrogen and progesterone may possibly have a beneficial role in females. Testosterone is known to promote atherosclerosis and retain sodium. It has been shown to affect the vascular tone.[26] Human RPE cells are found to have androgen receptors. There is therefore a possibility that testosterone is somehow related to the etiology of ICSC. There are two case reports of patients developing ICSC while on exogenous testosterone therapy.[26,27] The study by Haimovici *et al*,[28] was the only one where serum testosterone levels were estimated in patients of ICSC. The serum levels were found to be normal in 23 of the 24 cases. It is interesting to note that we have for the first time estimated serum testosterone levels in patients suffering from ICSC and compared them with age and sex-matched controls. The mean serum levels of testosterone were observed to be within the normal range in both ICSC cases and controls. This finding is in conformity with those of the study referred above.[28] We, however, found that about 32% patients of ICSC and 18% controls had lower than normal levels of testosterone.

We did not observe any significant correlation between levels of serum testosterone and the number and site of leaks on FFA. The small sample size in the present study, however, does not permit us to draw any conclusions regarding the role of testosterone in the pathogenesis of ICSC. Further large-scale studies investigating the probable role of testosterone levels in ICSC need to be carried out.

To conclude, it may be logical to believe that ICSC remains a challenge to the ophthalmologist as the exact etiopathogenesis remains poorly understood in spite of tremendous clinical and experimental research. ICSC is associated with elevated 8.00 a.m. serum cortisol levels. Association of serum testosterone levels with ICSC needs to be further investigated.
